# Sea Bass Immunization to Downsize the Betanodavirus Protein Displayed in the Surface of Inactivated Repair-Less Bacteria

**DOI:** 10.3390/vaccines7030094

**Published:** 2019-08-20

**Authors:** Raquel Lama, Patricia Pereiro, Beatriz Novoa, Julio Coll

**Affiliations:** 1 Institute of Marine Research (IIM), Spanish National Research Council (CSIC), Eduardo Cabello 6, 36208 Vigo, Spain; 2 Biotechnology Department, National Institute for Agricultural and Food Research and Technology (INIA), La Coruña road, 28040 Madrid, Spain

**Keywords:** VNNV, mass-immunization, sea bass, recombinant bacterins, spinycterins, DNA-damaged, repair-less

## Abstract

This work describes immunization of European sea bass (*Dicentrarchus labrax*) juveniles against viral nervous necrosis virus (VNNV), a *betanodavirus* causing worldwide mortalities in many fish species. Protection was obtained with the so-called spinycterin vehicles consisting of irreversibly DNA-damaged DNA-repair-less *Escherichia coli* displaying at their surface a downsized VNNV coat antigen. In this work we have (i) maximized bacterial expression levels by downsizing the coat protein of VNNV to a fragment (frgC_91–220_) containing most of its previously determined antigenicity, (ii) developed a scalable autoinduction culture media for *E. coli* based in soy-bean rather than in casein hydrolysates, (iii) enriched surface expression by screening different anchors from several prokaryotic sources (anchor + frgC_91–220_ recombinant products), (iv) preserved frgC_91–220_ antigenicity by inactivating bacteria by irreversible DNA-damage by means of Ciprofloxacin, and (v) increased safety using a repair-less *E. coli* strain as chassis for the spinycterins. These spinycterins protected fish against VNNV challenge with partial (Nmistic + frgC_91–220_) or total (YBEL + frgC_91–220_) levels of protection, in contrast to fish immunized with frgC_91–220_ spinycterins_._ The proposed spinycterin platform has high levels of environmental safety and cost effectiveness and required no adjuvants, thus providing potential to further develop VNNV vaccines for sustainable aquaculture.

## 1. Introduction

Viral encephalopathy and retinopathy cause up to 100% mortalities in juveniles of more than 40 finfish species including those most important to the European marine aquaculture industry such as sea bass (*Dicentrarchus labrax*) and sea bream (*Sparus aurata*) [1,2]. All these diseases are caused by viral nervous necrosis viruses (VNNVs) which belong to the *Nodaviridae* family within the *betanodavirus* genus [3,4]. VNNVs are non-enveloped particles of icosahedral symmetry enclosing two single-stranded, positive sense RNAs. One of the RNAs encodes an RNA-dependent RNA polymerase, while the other encodes their coat protein (C protein). According to C gene-derived protein sequences, *betanodavirus* isolates from Europe, Asia and Japan could be classified into 4 genotypes, but displaying only 19–23% differences among them [5,6]. Most C proteins of geographically-related betanodaviruses share up to 98–99% of their amino acid sequence.

Different types of VNNV killed vaccines have been described [7], including those made with inactivated virus [8,9], VLP virus-like particles [10,11,12], recombinant C proteins [13,14], or synthetic peptides derived from the C protein [15]. Most of those have to be delivered by fish-to-fish injection such as intraperitoneal injection of formalin-inactivated betanodaviruses [16]. Thus, an oil-adjuvanted intraperitoneal injectable vaccine that protects 12 g sea bass against the RGNNV genotype for one year has been available for emergencies since 2014 and received market authorizations in 2018 in Spain, Italy, Croatia and Greece (https://www.pharmaq.no/updates/pharmaq-has-rec/). Alternative innovative vaccination immersion protocols have been described for sea bass [17], and specific antibodies were induced in grouper eggs by vertical transmission from broodfish injected with inactivated VNNV [18]. Vaccination methods against nodaviruses and their corresponding immune responses in European sea bass have been recently reviewed [19] including oral delivery alternatives such as those using inactivated bacteria encapsulating dsRNA from VNNV, and chitosan conjugated VNNV DNA [7]. Most recently, protection has been reported by using alive recombinant bacteria expressing the C protein sequence mixed with the feed [20]. Although the use of recombinant bacteria will be most appreciated for large scale oral vaccination by avoiding stressful, labour intensive and costly delivery, the release of alive genetically modified organisms (GMOs) will have practical problems. Thus, the presence of recombinant DNA and antibiotic resistance genes in alive or even in inactivated GMOs will raise safety concerns for sustainable aquaculture.

To investigate alternatives to live or dead recombinant bacteria, we have explored here a previously reported platform consisting of formaldehyde-inactivated recombinant bacteria displaying downsized viral antigens in their surface (called spinycterins) [21]. Such spinycterins were obtained by genetic fusion of selected prokaryotic anchor-motifs to the N-terminal part of small linear immunodominant viral fragments. Despite the high reduction of antigenicity caused by formaldehyde crosslinking, successful production of anti-viral antibodies were demonstrated by immersion of ultrasound-treated zebrafish and/or carps in spinycterins displaying downsized CyHV-3 herpesvirus [21]. Among the safety advantages, the spinycterin inactivated condition may allow also for lyophilization and/or addition into feeds, contributing also to bypass the low temperature-dependence of fish vaccines. However, several fine-tuning details need improvement to favor further development of spinycterins for small fish vaccines. First, there is no previous evidence that shows that any spinycterin displaying downsized VNNV antigens will induce protection against VNNV challenge [21]. Second, crosslinking by formaldehyde inactivation caused a ~80% antigenicity loss [21]. Third, the yields of expression of some the anchor fusions were low or inhibited bacterial growth [21]. Fourth, safety concerns may still remain when handling and releasing to the environment large amounts of recombinant bacteria and those need to be minimized even when using GMOs which may have some of their DNA intact despite inactivation. Therefore, improvements in the above-mentioned concepts were explored in spinycterins made with downsized VNNV antigens.

Because the fish host VNNV antigenicity is focused on its coat (C) protein, downsizing of the C protein was performed as a means to increase its expression levels in recombinant *E. coli* while maintaining the immunogenic potential of the antigen [22,23]. To provide for bacterial surface display, several prokaryotic membrane anchor-motifs were fused to the downsized C protein. The anchor-motifs employed in this work, included those used before [21] and the P9 anchor-motif identified in the envelope of phage ϕ6 [24]. Because of the importance of nodaviruses in the aquaculture of commercially important fish species such as sea bass and sea bream, we chose one of them (sea bass) to validate protection of spinycterins against the VNNV challenge.

To preserve the initial immunogenicity of recombinant bacteria in the resulting spinycterins, several alternative methods to formaldehyde inactivation were explored. Among the many alternatives described before, bactericidal drugs appeared to be an attractive possibility since they allow for 100% of preservation of antigenicity, while maintaining intact the recombinant bacterial morphology and their inherent adjuvanticity. Among the described bactericidal drugs, those that target double-stranded DNA, introducing stable breaks into its strands by covalent binding and causing dead by oxidative ROS (i.e., bacterial DNA gyrase inhibitors), were preferred in contrast to those targeted to bacterial DNA-dependent RNA synthesis (i.e., rifampicins), cell-wall envelopes (wall synthesis inhibitors), and/or bacterial protein translation (synthesis inhibitors) [25]. Therefore, we explored the possibilities of some of these drugs to irreversibly inactivate surface displaying bacteria in a cost-efficient manner preferably by damaging their double stranded DNA by introducing stable breaks.

In addition, to increase safety we explored the DNA-repair deficient BLR(DE3) strain. In contrast to the BL21(DE3) *E. coli*, the derived BLR(DE3) strain cannot repair double DNA strand breaks, nor revert antibiotic-dependent ROS oxidation damage, thus making their derived recombinants more susceptible to DNA inactivation methods [26]. Additionally, the BLR(DE3) strain is resistant to tetracycline (TetR) which is more convenient for large-scale manufacturing because it makes possible to reduce any possible contaminant bacterial growth. Furthermore, BLR(DE3) requires Isoleucine (Ileu^-^) in the culture media to grow [27], opening the possibility to develop antibiotic-independent recombinant selective methods to reduce the possibilities to spread resistant genes. All these characteristics make BLR(DE3) highly advantageous for large-scale production, but it is not yet known whether BLR(DE3) can be used to produce spinycterins.

The results obtained in this work showed that a new bacterial culture media containing soy-bean rather than casein hydrolysates made anchor-antigen expression more reproducible by delaying autoinduction and eliminating the IPTG requirement. In addition, Ciprofloxacin inactivation irreversibly damaged the DNA of the recombinant bacteria by covalent binding to the DNA strands and killed the bacteria by oxidative ROS mechanisms while preserving all antigen immunogenicity, therefore increasing safety during both manipulations and delivery of the resulting spinycterins. Finally, BLR(DE3) was a good substitute for BL21(DE3), adding another safety level. Because of all these properties, irreversibly DNA-damaged recombinant BLR(DE3) displaying downsized viral antigens may be used to further develop new adjuvant-less spinycterin vehicles for VNNV antigens in an environmental safer way. These spinycterins may not only contribute to move ahead the state-of-the art of small fish viral vaccinology but also other veterinary vaccination procedures.

## 2. Materials and Methods

### 2.1. Construction of Downsized VNNV Coat Sequence and Genetic Fusion to Prokaryotic Anchor-Motifs

The frgC_91–220_ sequence (amino acid residues 91–220) was derived from the C protein of viral nervous necrosis virus (VNNV) isolate AY284959 from *D. labrax* [22,23]. The C protein-derived sequence was fused downstream of 6 different bacterial membrane anchor-motif sequences (Table 1). The different constructs were genetically fused by arbitrary flexible linkers (corresponding to the amino acid sequence GlicSerGliSer, GSGS). All the corresponding DNA sequences were chemically synthesized (GeneArt, Regensburg, Germany) and subcloned in between the NdeI and HindIII restriction sites of the pRSET prokaryotic expression plasmid which adds poly-histidine tails (polyH) at the C-terminal end of its multiple cloning site. Therefore, the general formula of the resulting recombinant constructs was: NH_2_-anchor + GSGS + frgC_91–220_ + GSGS + polyH-COOH. The purified plasmids were then transfected by the CaCl_2_ method into BL21(DE3) or BLR(DE3) *E. coli* strains and grown in either TB or SB media (Table 2) at 37 °C.

### 2.2. Induction of Protein Expression and Inactivation of Antigen Surface-Displaying Recombinant Bacteria

The BL21(DE3) *E. coli* strain has been widely used for recombinant protein expression including previously reported spinycterins for fish immunization to CyHV-3 herpesvirus [21]. The BLR (DE3) is a DNA-repair deficient transposon derivative strain of the BL21(DE3), lacking recombinase A (RecA^−^) [28], having tetracycline resistance and requiring isoleucine for growth [27] (F_ ompT hsdSB(rB_ mB_) gal dcm (DE3) D(srl _ recA)306:Tn10 (TetR)) (Novagen). To produce spinycterins, BL21(DE3) or BLR(DE3) recombinant bacteria were grown overnight by strong agitation at 37 °C in 40 mL of TB or SB medium (see composition in Table 2) with 100 µg/mL of ampicillin for the BL21(DE3) strain or 100 µg/mL ampicillin, and 12.5 µg/mL tetracycline for the BLR(DE3) strain. To induce recombinant protein expression, either 0.5 mM IPTG was added every 2 h twice in TB media or autoinduction was allow to proceed during 4 days in SB media. The resulting bacteria were washed in PBS, and adjusted to a final concentration of 10^10^ cfu/mL. To irreversibly damage their DNA, Ciprofloxacin was added at 50 µg/mL, and incubated at room temperature for 2 h with agitation. The resulting spinycterins were finally washed with PBS, 20% glycerol was added to preserve bacterial morphology, followed by storage at −20 °C until use.

### 2.3. Characterization of the Expression of pRSET-Anchor + frgC_91–220_ Coded Proteins

Confirmation of anchor + frgC_91–220_ + polyH expression was performed in spinycterin pellets boiled in buffer containing SDS and β-mercaptoethanol. The proteins separated in gels (SDS-precast 4–20% polyacrylamide gels from BioRad, Richmond, Vi, USA) were either stained by Coomassie-blue or transferred to nitrocellulose membranes (BioRad) for Western blotting. For blotting, the membranes were blocked with dilution buffer (0.5% bovine serum albumin, 5% of skim milk, 0.1% Tween-20, 0.01% merthiolate, 0.005% phenol red in phosphate buffered saline pH 6.7). Membranes were then incubated with peroxidase labeled anti-polyH monoclonal antibody (Sigma Che. Co, St.Louis, Ms, USA). Bands were visualized using diaminobenzidine DAB stain.

### 2.4. Assay of anchor + frgc_91–220_ + polyh Enrichment on the Bacterial Surface

To assay the anchor + frgC_91–220_ + polyH enrichment, the spinycterins were first “shaved” by partial digestion with 1% of trypsin during 2 h at 37 °C. Control undigested spinycterins were incubated in parallel with PBS. Trypsin-dependent digestion levels were then estimated by polyacrylamide gel electrophoresis (PAGE) and by enzyme linked immunosorbent assays (ELISA).

To assay by PAGE, the amounts of recombinant bands after Coomassie blue staining of PAGE were compared between trypsin-digested and control undigested spinycterins. The resulting Coomassie-stained PAGE bands were densitometrized by Image J 1.41o (http://rsb.info.nih.gov/ij). The percentage of the recombinant protein bands relative to the total protein bands was first normalized by the formula, optical density of the recombinant anchor + frgC_91–220_ + polyH band/total optical density of the corresponding spinycterin proteins. The optical densities were then expressed relative to the optical density of frgC_91–220_ spinycterins by the formula, 100 × % of the anchor + frgC_91–220_ + polyH/% of frgC_91–220_ + polyH.

To assay by ELISA, several dilutions of the trypsin-digested and control undigested spinycterins were used to coat wells of polystyrene plates of Maxisorb 96-wells by overnight incubation to dryness at 37 °C. The coated solid-phases were then blocked by overnight incubation with 100 μL per well of dilution buffer (0.5% bovine serum albumin, 0.1% Tween-20, 0.01% merthiolate, 0.005% phenol red in phosphate buffered saline pH 6.7) containing 10 µg per well of skimmed milk (Sigma, Che. Co.). After washing, the wells were incubated with peroxidase-labeled anti-polyH monoclonal antibody (Sigma Che. Co.) in 50 µL per well during 60 min. After washing 3 times, the colour reaction was developed by adding 50 µL of 1 mg/mL o-phenylenediamine in citrate buffer containing 3 mM H_2_O_2_. Absorbances were measured by reading at dual-wave lengths at 492–620 nm to correct for individual differences between wells. PolyH binding was calculated in percentage by the formula, 100× Absorbance after trypsin digestion/Absorbance of undigested samples. The percentage of absorbance was then expressed relative to frgC_91–220_ spinycterins by the formula, 100 × absorbance of the anchor + frgC_91–220_ + polyH/absorbance of frgC_91–220_ + polyH.

### 2.5. Immunization with Surface-Displaying Bacterins by Intraperitoneal Injection

Groups of 15–20 fingerling sea bass (*D. labrax*) of ~10 g of body weight were acclimatized for 30 days to sea water aquaria closed-circuits of 60 L each. Fish from two independent aquaria for each spinycterin antigen were injected with 100 µL of PBS (two independent intraperitoneal injections of 50 µL each per fish) containing 10^8^ cfu of frgC_91–220_ + polyH, Nmistic + frgC_91–220_ + polyH or YBEL + frgC_91–220_ + polyH spinycterins. Non-infected, infected with the empty pRSET plasmid and non-immunized controls were also included. The fish were fed with commercial pellets and maintained at 26 °C during 30 days. Viral challenge was performed by intramuscular injection of 100 µL of the VNNV strain 475–9/99 (2 × 10^4^ TCID_50_ per mL) of the RGNNV genotype provided by the Instituto Zooprofilacttico delle Venize (Italy) isolated from *D. labrax* [29]. Mortalities were recorded daily. Relative percentage of survival was calculated by the formula, 100–percentage of mortality in the spinycterin-immunized VNNV-challenged fish/percentage of mortality in the non-immunized VNNV-challenged fish.

### 2.6. Ethic Statement on Fish Handling

Fish care and challenge experiments were reviewed and approved by the CSIC National Committee on Bioethics under approval number ES360570202001/16/FUN01/PAT.05/tipoE/BNG, following the National Guidelines for type III experimentation (Annex X, permission RD53/2013) and the EU directive 2010/63/EU for animal experiments (http://ec.europa.eu/environment/chemicals/lab_animals/legislation_en.htm). To record possible mortalities, the immunized fish were monitored daily 2-4 times during 1 month. Methanesulfonate 3-aminobenzoic acid ethyl ester (MS222) was used to euthanize moribund fish by an overdose of MS222 (200 mg/mL) to minimize suffering.

## 3. Results

### 3.1. Selection of the frgc_91–220_ Sequence from the Betanodavirus C Coat Protein

**Figure 1:**
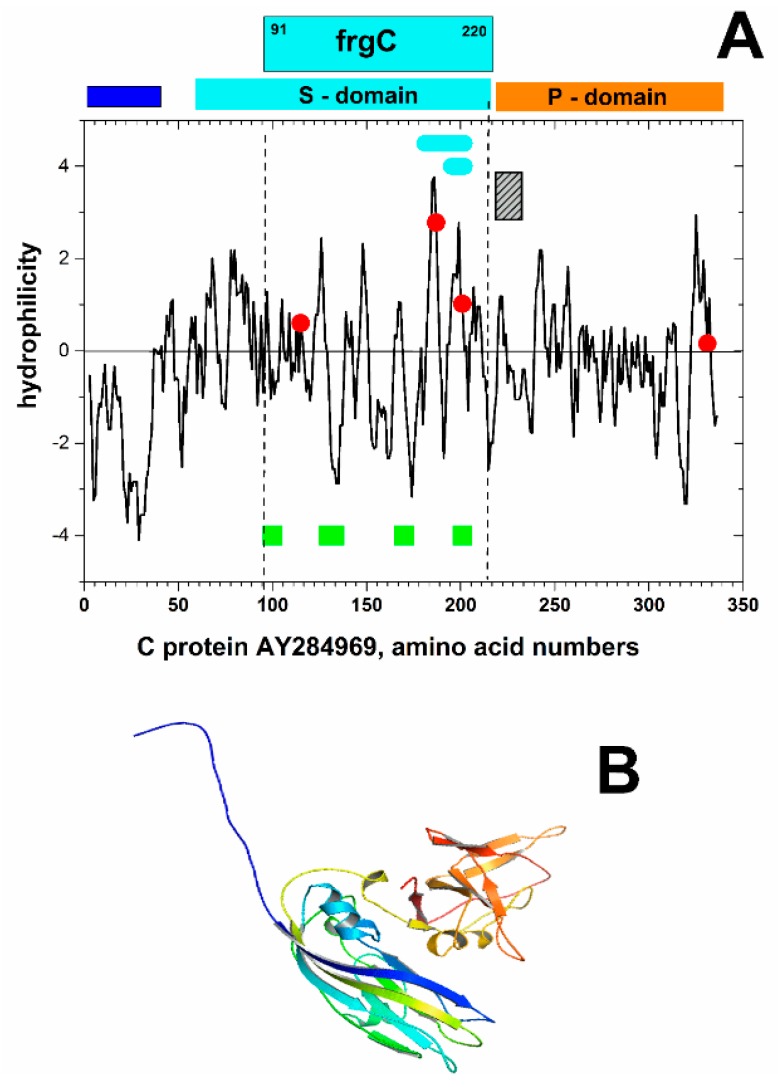
Scheme of the hydropathicity (**A**) and tridimensional structure (**B**) properties of frgC_91–220._

To select an immunorelevant VNNV antigen for sea bass vaccination, the C-coat protein was chosen because it is the only target for fish neutralizing antibodies. Since a minimal antigen size favors expression on *E. coli* taking into account the additional increment of molecular size because of the fusion with a prokaryotic membrane anchor required for surface display, we studied the best way to downsize the C protein. Since targeted epitopes of both neutralizing monoclonal antibodies and serum samples from VNNV-infected survivor fish were mapped at amino acid positions 1–32, 91–162 and 181–212 [23,30], the fragment extending from the 91 to the 220 amino acid was chosen for optimal antigenic expression on *E. coli*. The frgC_91–220_ contained most of the C shell domain (S-domain) located between amino acid residues 52–213 [31] (Figure 1A,B), the highest hydrophilic regions of exposed amino acid residues on the viral surface (Figure 1A), three cysteines, all the Ca++ binding sites implicated in subunit-subunit C-interactions in the viral shell structure [31] and most of the neutralizing B-cell epitopes mentioned above [23,30]. By choosing the frgC_91–220_, we avoided the highly hydrophobic 1–32 signal peptide which is deleterious to *E. coli*. We also avoided the 223–331 region that contains most of the sequence variability among VNNV isolates [5] providing a common immunogen for a wider number of VNNV isolates. Finally, the three cysteins were mutated to serines during the gene synthesis to avoid the formation of *E. coli* inclusion bodies with reduced-immunogenicity.

**Figure 2:**
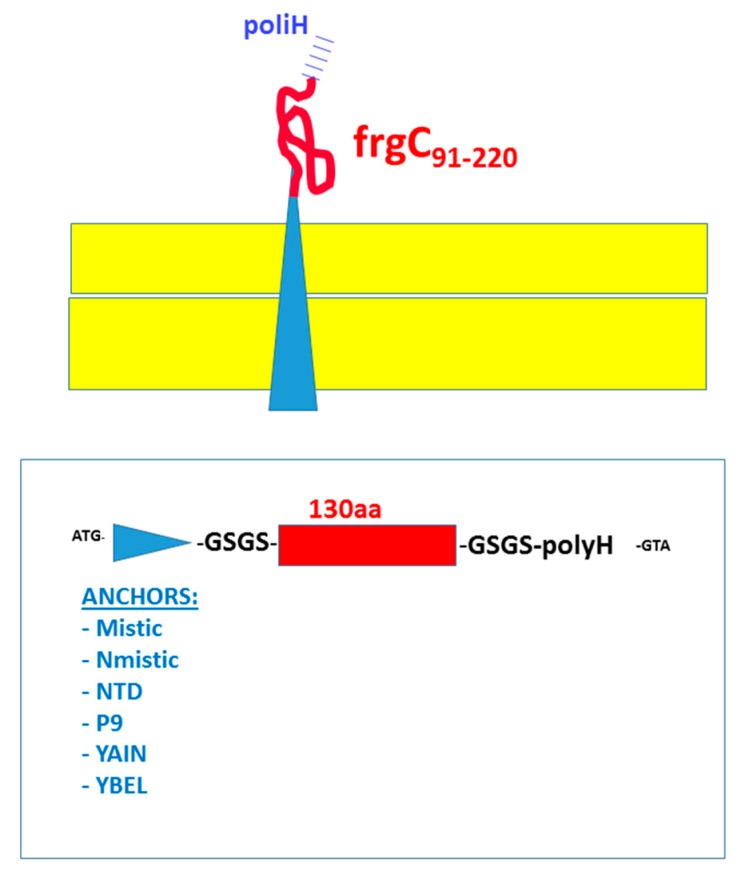
Scheme of the genetically fused constructs for bacterial surface expression of frgC_91–220._

To select for the best anchors to increase bacterial membrane expression, several prokaryotic anchor-motifs were genetically fused to frgC_91–220_. Those included Mistic, Nmistic, NTD, P9, YAIN and YBEL [24,32,33,34,35,36,37,38,39]. Most of these anchor-motifs were already described when fused to a CyHV-3 herpesvirus fragment [21], but the genetic fusion to P9 [24] is described here for the first time. The P9 protein of 90 amino acids constitutes the major envelope of phage ϕ6. It was proposed as a bacterial anchor alternative because it facilitated the integration of 11 of 14 target proteins into the *E. coli* cell membrane [24]. Therefore, the general formulas of the resulting recombinant constructs were NH_2_-prokaryotic membrane anchor-motif + GliSerGliSer (GSGS) linker + frgC_91–220_ + GSGS linker + polyHis(H)-COOH (Figure 2). The expected molecular weights of the recombinant products fused to the selected prokaryotic anchors varied from 20 to 34.8 KDa while the frgC_91–220_ was 16.2 KDa (Table 1).

### 3.2. Autoinduction Media to Improve Anchor-Fused FrgC_91–220_ Expression

Previous results revealed partial or total inhibition of expression of some of the anchors in the fused constructs with herpes viral proteins coded in pRSET plasmids under the control of T7/lactose promoters in BL21(DE3) *E. coli* [21]. Most probably the presence of lactose in the casein hydrolysates of LB/TB media contributed to an early autoinduction which resulted in toxicity during the growth phase, similarly to what has been reported in other T7/lac promoter expression systems [40,41]. To reduce early autoinduction, the casein hydrolysate broth (LB/TB) was substituted by soy-bean hydrolysate broth (SB). Because of the small amounts of galactose in SB, a weaker inducer than lactose, soy-bean hydrolysates may be used for delaying autoinduction to the stationary phase, thus increasing yields and reproducibility [40,42]. Furthermore because of its plant origin, the SB medium contains no animal infectious contamination, which is important for large-scale production and release into the environment. After numerous tests, the composition of the SB media for maximal and reproducible yields of autoinducible expression of anchored frgC_91–220_ resulted in the formula shown in Table 2.

**Figure 3:**
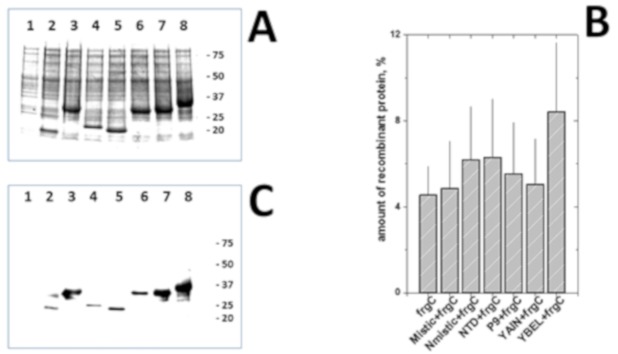
Coomassie-blue staining (**A,B**) and Western blotting (**C**) of anchor-motif+frgC_91–220_ spinycterins grown and autoinduced in SB medium.

*E. coli* BL21(DE3) clones transformed with each of the anchor+frgC_91–220_ pRSET plasmids were selected from SB agar plates in the presence of ampicillin and grown in 2 mL SB cultures. All the recombinant proteins fused to any of the 6 anchors were efficiently expressed in *E. coli* (Figure 3A), in contrast to the variability previously observed when grown in TB [21]. Densitometry estimations of each of the anchor + frgC_91–220_ proteins separated by polyacrylamide gel electrophoresis (PAGE) and identified by the expected molecular weights of their Coomassie-blue stained bands (Table 1) and western blots using anti-polyH antibodies (Figure 3C) varied between 4.1 to 8.4% (*n* = 3) of the total stained protein in each of the *E. coli* extracts, while the estimate of the frgC_91–220_ in the absence of any anchors was 4.5 ± 1.3% (Figure 3B). The YBEL+frgC_91–220_ spinycterins showed the highest expression level (8.4 ± 3.2%).

We then selected one of the lower and the higher molecular weight constructs (Nmistic and YBEL, respectively) to scale up production for additional testing. The results confirmed that slightly lower but similar yields of recombinant products could be produced in 40 mL cultures. Thus, 2.4 ± 0.7%, 2.7 ± 0.3 and 4.6 ± 2.1% of stained protein were estimated for the frgC_91–220_, Nmistic + frgC_91–220_ and the YBEL+frgC_91–220_ constructs, respectively (not shown).

### 3.3. Inactivation of Recombinant E. coli by DNA-Damage

**Figure 4:**
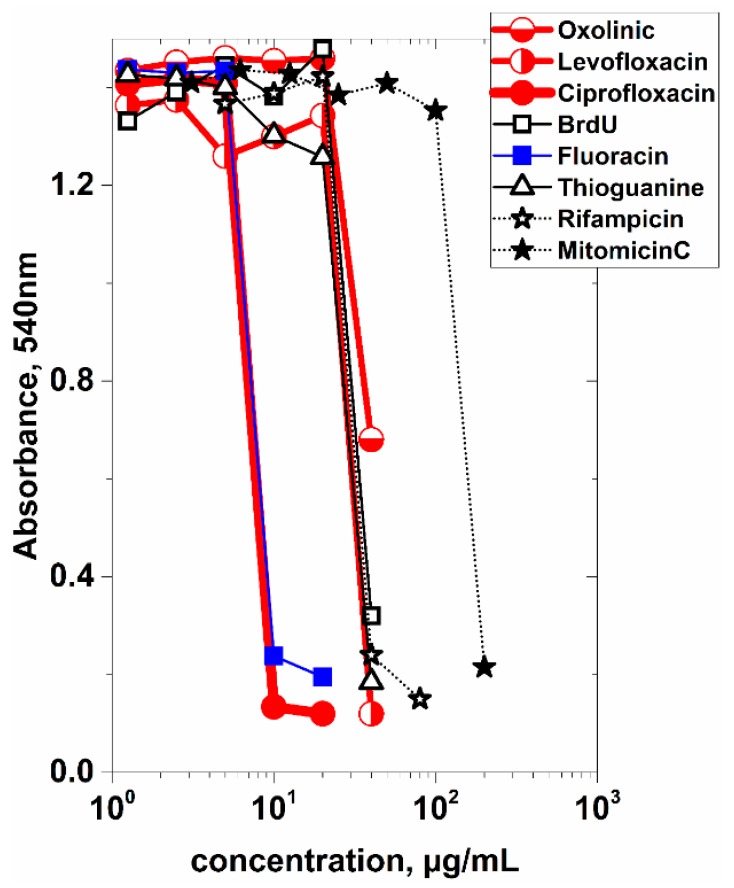
Selection of drugs for inactivation of recombinant *E. coli* BL21(DE3).

To be safely handled and released to the environment, the bacteria expressing the anchor + frgC_91–220_, need to be irreversibly inactivated. However, alternative inactivation methods were needed to improve the loss of antigen reactivity shown before by crosslinking with formaldehyde [21]. Preliminary experiments performed with YBEL + frgC_91–220_ BL21(DE3) *E. coli* showed no colony formation after treatment with Ciprofloxacin and Leucofloxacin at the lowest 20 µg/mL concentration tested in contrast to Oxolinic acid, Rifampicin and controls without any treatment (Figure S1A). Furthermore, no significant changes in the level of expressed YBEL + frgC_91–220_ recombinant protein were observed when the spinycterin extracts were separated by polyacrylamide gel electrophoresis stained with Coomassie-blue (Figure S1B). After exploring these results drugs damaging the bacterial DNA appeared among the most convenient since they inhibited bacterial colony formation at low concentrations preserving recombinant protein levels). To select for a suitable drug to irreversibly damage the DNA of *E. coli*, several selected antibiotics and/or base analogs (Oxolinic acid, Levofloxacin, Ciprofloxacin, BrdU, Fluoracin, Thioguanine, Rifampicin, and Mitomicin C) were screened at different concentrations for inhibition of replication of YBEL+frgC_91–220_ spinycterins made in BL21(DE3) *E. coli*. According to the corresponding survival concentration-dependent curves, the most active compounds were Ciprofloxacin (CPFX) and 5-Fluoracin (Figure 4). CPFX was selected for further studies because it cleaves and link to DNA strands and causes oxidation-related bactericidal effects. In addition, CPFX was easily available at a low price and in contrast to some of the other drugs, up to 1600 µg per mL did not affect the levels of recombinant protein expression, as estimated by Coomassie-blue staining of the resulting spinycterin extracts after overnight incubation (not shown).

### 3.4. Recombinant Protein Expression Levels Were Lower when Using the Dna-Repair Deficient blr(de3) E. coli Strain

**Figure 5:**
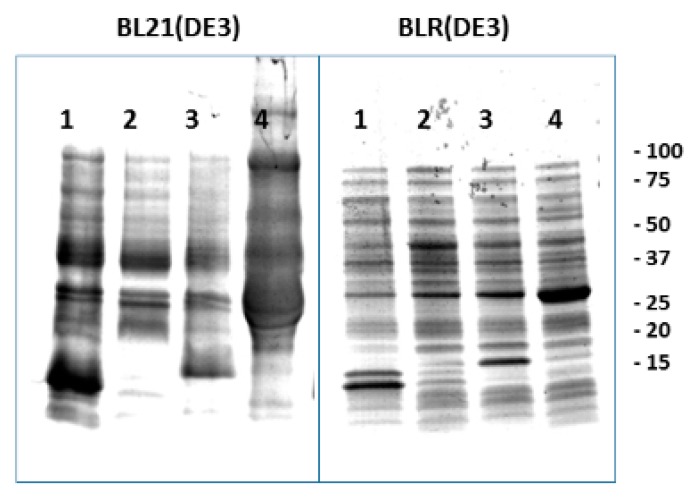
Coomassie-blue staining of polyacrylamide gel electrophoresed anchor-motif + frgC_91–220_ spinycterins obtained in large amounts in BL21(DE3) and BLR(DE3) *E. coli* strains.

To compare the expression levels of the recombinant display constructs in the BL21(DE3) and the repair-less BLR(DE3) *E. coli*-derived spinycterins, both were grown in parallel 40 mL cultures. Results showed a higher expression level in comparison with the 2 mL cultures (Figure 5). However, while the frgC_91–220_ was similarly expressed in the two *E. coli* strains (14.3 ± 4.5% and 12.3 ± 3.7%, respectively, *n* = 2), the expression level of the corresponding Nmistic + frgC_91–220_ and YBEL + frgC_91–220_ was lower when produced in the BLR(DE3) than in the BL21(DE3) strain (5.4 ± 0.4 and 12.4 ± 0.5, respectively, in BLR compared to 17.7 ± 0.2 and 36.3 ± 5.4, respectively, in BL21, *n* = 2).

To test for the irreversibility of inactivation by the CPFX treatment, the BLR(DE3) Nmistic+frgC_91–220_ or YBEL+frgC_91–220_ bacteria were treated with several concentrations of CPFX and the viability of the resulting spinycterins tested by both overnight growth in TB-ampicillin-tetracycline plates and several days overgrowth in 2 mL of TB-ampicillin-tetracycline medium. No colonies nor growth could be detected after treatment with 1 µg/mL of CPFX, in contrast to untreated bacteria with an initial inocula of 10^9^ cfu (not shown). This CPFX dosage was ~100-fold lower than the inactivation obtained for the BL21(DE3) strain (Figure 4), most probably due to the DNA-repair deficiency of the BLR(DE3) strain.

### 3.5. Surface Expression of Nmistic + frgC_91–220_ and YBEL + frgC_91–220_ in BLR(DE3) Spinycterins

**Figure 6:**
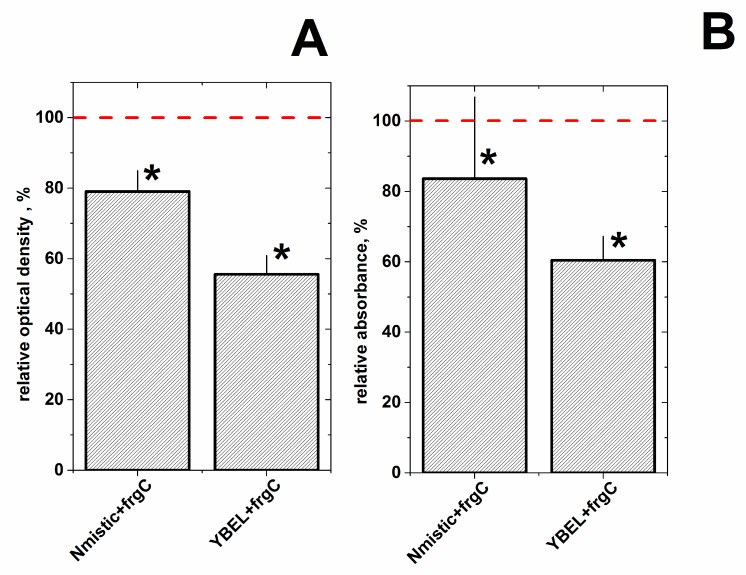
Estimation of frgC_91–220_ surface enrichment by partial trypsin digestion of BLR(DE3) spinycterins followed by PAGE (**A**) and ELISA (**B**) analysis.

To estimate the surface enrichment of frgC_91–220_ in the selected spinycterins, PAGE and ELISA experiments were carried out after partially “shaving” the corresponding spinycterin surfaces by limited trypsin digestion. The higher surface exposure of frgC_91–220_ should be more susceptible to trypsin digestion and therefore should show a stained-band of lower intensity by PAGE and a lower anti-polyH binding absorbance by ELISA. Surface exposure of frgC_91–220_ estimated by comparing the Coomassie-blue stained bands of anchor+frgC_91–220_ with those from frgC_91–220_ spinycterins showed that the most exposed levels were obtained for the YBEL + frgC_91–220_ spinycterins (55.5 ± 5.3% stained band intensity remaining after trypsin digestion) followed by the Nmistic + frgC_91–220_ (79.05 ± 5.8%) spinycterins (Figure 6A), suggesting that the YBEL + frgC_91–220_ had the highest surface exposure_._ The polyH binding of frgC_91–220_ to peroxidase labelled anti-polyH antibodies after trypsin digestion estimated by ELISA showed again that the best levels of surface expression were obtained for the YBEL + frgC_91–220_ spinycterins (60.4 ± 13.5% anti-polyH binding after trypsin digestion) followed by the Nmistic + frgC_91–220_ spinycterins (83.6 ± 40.1%) compared to frgC_91–220_ spinycterins (Figure 6B). Therefore, the results obtained by both methods confirmed that the surface exposure of frgC_91–220_ was higher in the YBEL+frgC_91–220_ than in the Nmistic+frgC_91–220_ BLR(DE3) spinycterins.

To explore whether additional paraformaldehyde-based crosslinking methods could be applied to inactivate spinycterins or to further inactivate CPFX-spinycterins, surface exposure of the anchor + frgC_91–220_ + polyH was estimated by anti-polyH binding after paraformaldehyde treatment. Results showed that while the polyH reactivity of bacterins expressing the Nmistic + frgC_91–220_ was nearly unaffected by the paraformaldehyde treatment, those expressing the larger molecular weight YBEL + frgC_91–220_ lost most of their anti-polyH binding (Figure S2B). These results showed that although alternative crosslinking methods, such as paraformaldehyde, could be used to inactivate spinycterins, these possibilities may depend on the anchor-motif employed for surface expression. The differences detected between the anti-polyH binding of the YBEL + frgC_91–220_ and the Nmistic + frgC_91–220_ BLR(DE3) spinycterins could be due to the larger content in primary amino-exposing lysine and arginine amino acids per mol of the highly hydrophilic YBEL anchor compared to the Nmistic anchor (7 and 14 compared to 4 and 1, respectively).

### 3.6. In Vivo Protection Against VNNV Challenge Using Spinycterins Obtained in BLR(DE3) E. coli Displaying Anchor + frgC_91–220_

**Figure 7:**
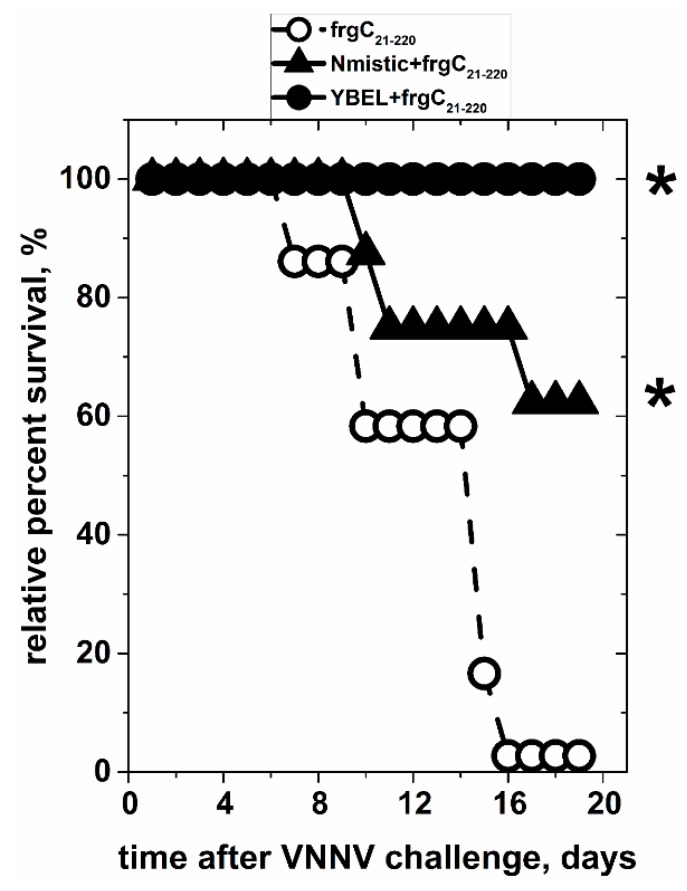
Protection to VNNV challenge of sea bass juveniles after intraperitoneal injection of frgC_91–220_, Nmistic + frgC_91–220_ or YBEL + frgC_91–220_ BLR(DE3) spinycterins.

The YBEL + frgC_91–220_ and the Nmistic + frgC_91–220_ BLR(DE3) spinycterins were intraperitoneally injected to fingerling sea bass to validate possible protection against VNNV challenge. Two independent aquaria were used to maintain the fish for each spinycterin injected. After the VNNV challenge, total mortalities obtained by adding the results from each of the two aquaria resulted in 25.7% mortality of the non-immunized but VNNV-challenged controls. Similar results were obtained for the fish injected with the empty pRSET plasmid. The protection level expressed in relative percent survival was 100% in fish injected with the YBEL + frgC_91–220_ spinycterins, while it was 62.3% in those injected with the Nmistic + frgC_91–220_ spinycterins_._ In contrast, a survival of 2.7% was obtained for those fish injected with the frgC_91–220_ spinycterins (Figure 7). A correlation between protection and surface exposure levels was suggested by comparing the above-mentioned results and those from Section 3.5.

## 4. Discussion

This work describes 100% protection of sea bass juveniles against VNNV challenge by spinycterin vehicles and a direct correlation between bacterial surface exposure and fish protection levels. The high level of protection was obtained in the absence of adjuvants with irreversibly DNA-damaged DNA-repair-less spinycterins. Furthermore, to our knowledge, this is the first time that inactivated bacteria displaying a recombinant cystein-free downsized C VNNVN-terminal antigen (frgC_91–220_) containing most of the epitopes targeted by fish neutralizing antibodies (B-cell epitopes) have been described for inducing protection against VNNV challenge. The frgC_91–220_ was fused to several prokaryotic membrane anchors to select the ones with higher membrane expression in *E. coli*. A correlation between the levels of bacterial surface expression and fish protection was demonstrated by comparing the corresponding data obtained with YBEL + frgC_91–220_ spinycterins (higher frgC_91–220_ surface display and full protection) with those of Nmistic + frgC_91–220_ (lower frgC_91–220_ surface display and partial protection) and frgC_91–220_ (lowest frgC_91–220_ surface display and lowest protection) spinycterins. These relatively high protection levels were obtained despite the selected frgC_91–220_ being located outside of the most important shell protrusion C-terminal domain (P domain, amino acids 214–338) in both VLP [43] and whole virus [31], which could had been expected to be more antigenic based only on predicted structural criteria (Figure 1B).

On the other hand, to improve spinycterin manufacturing, yields, reproducibility and safety, the following strategies were combined: a novel and scalable autoinduction soy-bean based media for *E. coli* expressing recombinant proteins under the control of the T7/lac promoter, inactivation through an irreversible DNA-damage alternative to traditional crosslinking inactivation, and a DNA repair-less *E. coli* strain as chassis.

A new auto-induction medium for BL21(DE3) *E. coli* culture was developed based on previous reports to reduce overexpression toxicity of some recombinant proteins [40,41,44]. Thus, we had previously found that the expression of some of the anchor-fusions to immunogenic proteins in *E. coli* under the T7/lac promoter control grown in bacterial culture media based on casein hydrolysates, such as LB or TB, were partially or totally inhibited, apparently due to toxicity during the growth phase [21]. Since such toxicity may be caused by early autoinduction of *E. coli* due to the presence of lactose in the casein hydrolysates during their fast growth rate [40], we undertook a series of experiments to reduce the residual lactose content of the culture media. Those experiments lead us to develop the so-called SB medium, a bacterial culture medium based on vegetable soy-bean rather than casein hydrolysates of animal origin. To further reduce autoinduction, glucose was also added to the media as previously recommended [40]. Using the SB media, the highest expression levels by PAGE/Western blotting were obtained for the YBEL + frgC_91–220_ construct among 6 other anchor-motif alternatives. Similar higher expression results were previously reported for YBEL + frgII_CyHV3_ when compared to other 6 anchor-motifs in spinycterins grown in TB media [21]. Although their growth rate was slower in SB medium, detectable levels of recombinant protein expression could be obtained for the 6 anchor-motifs studied (*E. coli*, *B. subtilis*, phage), in contrast to the problems with some of their yields previously obtained when using the TB medium [21]. The YBEL + frgC_91–220_ construct remained with the highest level of expression among the anchor-motifs studied when cultured in SB or TB media.

After the studies on the bacterial culture media, experiments were focused in finding inhibitors of DNA replication by searching for an alternative to crosslinking for bacterial inactivation which destroyed ~80% of the bacterial surface displayed frgII_CyHV3_ immunogenicity [21] or most of the anti-polyH binding of YBEL + frgC_91–220_ spinycterins (this work). Among the possible anti-bacterial drugs s screened for inactivation, those targeting DNA replication focused in their supercoiling steps, such as those belonging to the quinolone family, appeared to be the best alternative, thus quinolones target unwinding DNA gyrase or topoisomerase II (Gram-negative bacteria) and topoisomerase IV (Gram-positive bacteria) enzymes, by interacting with double stranded DNA, covalently binding to cleavaged DNA [45] and stopping strand rejoining during DNA replication [45]. Additional quinolone bactericidal irreversible effects are induced by the generation of harmful hydroxyl radicals or ROS [46]. The above-mentioned topoisomerases are essential in bacteria but absent in higher eukaryotes, making them an attractive possibility for the present purposes. The best studied gyrase is that from *E. coli*, which has A and B subunits. The A subunit cleaves and covalently binds DNA strands, while the B subunit rejoins the strands. Inhibition of further strand cleavage/rejoining by stabilisation of the covalent gyrase-DNA complex (gyrase poisoning) shows concentration-dependent bacteriostatic or bactericidal effects [45]. For instance, the Ciprofloxacin (CPFX) quinolone exhibits a bacteriostatic reversible activity at minimal concentrations and an irreversible bactericidal activity at higher concentrations [47,48]. First-generation quinolones derived from nalidixic/oxolinic acids are rarely used today because of their toxicity to eukaryotic cells. Second (i.e., Ciprofloxacin), third (i.e., Levofloxacin) and fourth (i.e., Gemifloxacin) generation quinolones are clinically used. After numerous experiments, Ciprofloxacin was selected for this work because of its high activity at low concentrations, its covalent linking to cleavaged DNA strands, the induction of irreversible DNA-damage (bactericidal) and its low cost. Because this is an area of intensive research, new quinolones may appear in the future to cause irreversible DNA-damage of recombinant bacteria with even lower concentrations which will reduce possible concerns about the use of antibiotics to inactivate bacteria. Among the reasons mentioned above, CPFX was preferentially chosen because it cleaves the double-stranded DNA by covalently linking itself to the DNA strands [45] in a related mechanism to that of formaldehyde/paraformaldehyde crosslinking. The low concentration required and the final washing steps that remove any excess of CPFX, reduces the possibility of free CPFX being released to the environment, very much like it does with crosslinked fish vaccines. In addition, CPFX induces a ROS-dependent bacterial killing effect without affecting antigenicity, in contrast to crosslinking [21]. To enhance safety, it may be possible to add formaldehyde/paraformaldehyde at low concentrations to some the spinycterins or to the CPFX-inactivated spinycterins, provided a crosslinking-resistant anchor-motif is used (for instance, in the case of paraformaldehyde with Nmistic+frgC_91–220_ spinycterins). Therefore, in the case the use of CPFX may be rejected because of being an antibiotic, other linking/crosslinking compounds could be further explored as alternatives for spinycterin inactivation. In each particular combination of anchor-motif and immunogen care should be taken as to preserve surface display and antigenicity. In this work, we have focused on describing a minimal proof-of-concept prototype of a fish vaccine alternative platform which should be further studied by other inactivation procedures, alternative mass delivery techniques and including host innate and adaptive immune responses. In its present state-of-the-art, the described spinycterin adjuvant-less vehicles require further work to be practical.

Even though some fish vaccines based in eukaryotic expression plasmids (i.e., DNA vaccines) have been recently approved, their use is still highly controversial in Europe [49]. Therefore, using immune-relevant viral protein antigens rather than DNA may still be an alternative. Furthermore, protein antigens coded in prokaryotic rather than eukaryotic plasmid vectors (like those employed for DNA vaccines), offer safer environmental possibilities. Both of the above commented properties and the maintenance of bacterial morphology in the spinycterins described here for substituting oil-adjuvants- [50,51,52,53] allow easier mass delivery and lower production costs. Because of the lack of cysteins in the displayed antigen, the spinycterins described here may be also looked as a method to reduce the generation of low-immunogenicity inclusion bodies during manufacturing very often found when expressing whole heterologous proteins in recombinant bacteria. Although recent results suggested that isolated nanopellets derived from bacterial inclusion bodies may also be immunogenic [54], their practical use would need additional purification steps, losing their bacterial morphology and their adjuvant properties. Most probably, the use of inclusion bodies as fish vaccines will require too high concentrations. Recently, however, intact recombinant bacteria carrying their inherent adjuvanticity and coding for the whole C VNNV protein have been reported to induce partial protection against VNNV together with very low levels of antibodies when orally delivered to sea bass [20]. Furthermore, injection of extracts corresponding to 10^10^ cfu of such recombinant bacteria per fish fully protected against VNNV challenge [20]. In this context, there are some practical advantages of the DNA-damaged recA- *E. coli* alternative described here when coding for surface-displayed downsized viral antigens (spinycterins) in comparison with the recombinant wild-type *E. coli* coding for the whole C VNNV protein [20]. For instance, as shown in this work, the injection of only 10^8^ cfu per fish of morphologically intact YBEL + frgC_91–220_ spinycterins fully protected against VNNV challenge in the absence of any adjuvants. Furthermore, spinycterins may be better accepted in aquaculture because they are safer due to their DNA-repair deficient *E. coli* vehicle. Other advantages may be due to the antigen downsizing concept providing a higher immunorelevant epitope density for a given mass of bacteria, and the future possibility to use spinycterins expressing mixes of different pathogen antigens in a single delivery. Additionally, because of its isoleucine deficiency, the BLR(DE3) *E. coli* strain opens up the possibility of future developing of antibiotic-free selection methods to eliminate any antibiotic resistance sequences from the vaccine vehicles and thus further increase their environmental safety.

AccNum, Gene Bank accession numbers. FrgC_91–220,_ amino acid residues 91–220 from the C coat protein of viral nervous necrosis virus VNNV (sequence accession number AY284959) [22,23]. The anchor-motif + GSGS + frgC_91–220_ + GSGS + polyH DNA sequences were designed, synthesized, cloned into pRSET, used to transform *E. coli* and autoinduced in SB medium. KDa, expected molecular weight of the recombinant proteins. Mistic and N-mistic, 110 and 33 N-terminal amino acids anchor-motif from the Mistic gene from *Bacillus subtilis.* NTD, N-terminal domain of 21 amino acids anchor-motif of the exosporal BclA protein from *Bacillus anthracis*. P9, 90 amino acid anchor-motif from the coat-protein of bacteriophage ϕ6. YAIN, 91 amino acid anchor-motif from the hydrophilic regulatory protein of the frmR operon of *E. coli* YBEL, 160 amino acid anchor-motif from the hydrophilic HTH-type transcriptional regulator DUF1451 family protein from *E. coli*.

(A) Hydropathicity plot of the coat protein C from the *D. labrax* encephalitis virus isolate DL-040899-IL (AY284969) obtained using Clone Manager vs 9. Shell (S, blue rectangle) and protrusion (P, red rectangle) domains were located at amino acid residues 52–213 and 221–338, respectively, according to X-ray data [31]. Blue top rectangle, frgC_91–220_ (amino acid residues 91–220). Blue horizontal lines inside the plot, neutralizing B-cell epitopes localized by pepscan mapping targeted by sera from sea bass surviving VNNV infection and by anti-VNNV neutralizing monoclonal antibodies [23] or by alanine-scanning mutagenesis [22]. Red circles, cysteine positions which were mutated to serines in the recombinant frgC_91–220_. Green squares, Ca^++^ binding sites for subunit-subunit interactions in the betanodavirus shell structure [31]. Grey hatched rectangle, highest protein sequence variability among betanodavirus isolates corresponding to the 223-331 amino acid positions [5]. (B) Scheme of the tridimensional structure of the C protein of the AY284969 isolate. The automatically predicted modelled structure of the C protein of the AY284969 isolate (using the Swiss model server), selected the 4WIZ.3.A sequence as the best template. The template was derived from a Grouper Nervous Necrosis Virus isolate [31] with a 99.11% of amino acid sequence identity.

The nucleotide sequences corresponding to the frgC_91–220_ of VNNV were fused downstream to each of the 6 bacterial membrane anchor-motif sequences described in Table 2, bracketed by an arbitrarily chosen flexible linker (coding for amino acids GliSerGlicSer, GSGS). All the corresponding synthetically fused DNA sequences (GeneArt, Regensburg, Germany) were cloned into the multiple cloning site of the pRSET prokaryotic expression plasmid using the NdeI and HindIII restriction sites and adding 6 Histidine tails (polyH) at their C-terminal ends. Red, FrgC_91–220_. Blue triangles, Anchor-motifs. Yellow, schematic bacterial membrane. Blue lines, C-terminal polyH tail.

(A) BL21 (DE3) *E. coli* coding for anchor-motif+frgC_91–220_ + polyH recombinant proteins were grown in autoinduction SB medium overnight. Bacteria were pelleted and their extracts analysed by Coomassie-blue staining. One representative experiment is represented. (B). Densitometry of the anchor-motif+frgC_91–220_ recombinant bands stained by Coomassie by Image J 1.41o software (http://rsb.info.nih.gov/ij). Means and standard deviations are shown (*n* = 3). (C) Western blotting of gels transferred to nitrocellulose membranes, stained with peroxide-labeled anti-polyH monoclonal antibody and visualized with DAB as described [21]. One of 3 experiments was represented. Numbers to the right of the gels, KDa positions of molecular weight markers. The anchor-motifs of the recombinant *E. coli* in A and C corresponded to lanes: 1, empty plasmid. 2, frgC_91–220_. 3, Mistic + frgC_91–220_. 4, Nmistic + frgC_91–220_. 5, NTD + frgC_91–220_. 6, P9 + frgC_91–220_, 7, YAIN + frgC_91–220_. 8, YBEL + frgC_91–220_.

To select for a suitable drug to inactivate *E. coli* BL21(DE3) without altering their immunogenicity, several antibiotics and/or base analogs were tested for YBEL + frgC_91–220_ spinycterin replication. The recombinant bacteria (3 × 10^10^ cfu/mL) were exposed overnight to several concentrations of the selected compounds in 150 µL of TB with continuous agitation. After 2 washes with PBS, 10 µL of the suspensions were inoculated into 100 µL of fresh TB medium. Bacterial growth was estimated by absorbance at 540 nm after overnight incubation with agitation at 37 °C. Upper-half open red circles, Oxolinic acid. Left-half open red circles, Levofloxacin. Solid red circles, Ciprofloxacin. Open squares, 5-Bromo deoxiuridine. Solid blue squares, 5-Fluoracin. Open triangles, 6-Thioguanine. Open stars, Rifampicin. Solid starts, Mitomicin C.

*E. coli* coding for anchor-motif + GSGS+frgC_91–220_ + GSGS + polyH recombinant proteins was obtained in either BL21 (DE3) or in the repair-deficient *recA-* BLR(DE3) *E. coli* strains and grown in SB medium overnight. The *E. coli* BL21 (DE3) were grown at 37 °C and induced with IPTG at 24 °C for 2 h. The *E. coli* BLR(DE3) were grown and autoinduced for additional 4 days at 37 °C. The resulting suspensions were incubated at 24 °C for 2 h with 125 µg/mL of Ciprofloxacin (CPFX) for irreversible inactivation to generate spinycterins. Extracts were analysed as described in Figure 3. One of 2 experiments was represented. Numbers to the right of the gels, KDa positions of Coomassie blue stained molecular weight markers. Lanes: 1, frgC_91–220_ spinycterins. 2, empty pRSET plasmid spinycterins. 3, Nmistic + frgC_91–220_ spinycterins. 4, YBEL + frgC_91–220_ spinycterins.

(A) To assay by polyacrylamide gel electrophoresis (PAGE), the amounts of the corresponding stained bands were compared between trypsin-digested and control undigested BLR(DE3) spinycterins. The optical densities were first normalized by the formula, optical density of the recombinant bands/total optical density of each spinycterin extract. The normalized optical densities were then calculated relative to the optical density obtained in the frgC_91–220_ spinycterin bands by the formula, 100 × (% of anchor + frgC_91–220_/% of frgC_91–220_). Means and standard deviations (*n* = 3) were represented. (B) To assay by ELISA, 96-well plates were coated with trypsin-digested or control undigested BLR(DE3) spinycterins. The amount of exposed polyH tails was estimated by binding to peroxidase-conjugated anti-polyH monoclonal antibodies. Percentage of polyH-binding was calculated by the formula, 100 × (Absorbance after trypsin digestion/Absorbance of undigested samples). The percentage of absorbance was then calculated relative to the frgC_91–220_ spinycterins by the formula, 100 × (absorbance of anchor + frgC_91–220_/absorbance of frgC_91–220_. Means and standard deviations (*n* = 3) are presented. Red horizontal dashed lines, mean optical density (A) and absorbance (B) of frgC_91–220_ spinycterins *, significatively different from frgC_91–220_ spinycterins as determined by the Student t-test (*p* < 0.05).

Two independent aquaria per group, each containing 15–20 fingerling sea bass (*D. labrax*) of ~10 g of body weight were injected with 10^8^ cfu of frgC_91–220_, Nmistic + frgC_91–220_ or YBEL + frgC_91–220_ spinycterins. Non-infected, injected with empty pRSET plasmid and non-immunized controls were also included. Viral challenge was performed by intramuscular injection of VNNV (2 × 10^4^ TCID_50_/_mL_). Mortality in the non-immunized VNNV-challenged group controls was 25.7%. Relative percentages of survival were calculated by the formula 100–(percentage of mortality in the spinycterin-immunized VNNV-challenged fish/percentage of mortality in the non-immunized VNNV-challenged fish). *, significantly different from the frgC_91–220_ survival by the Log-Rank (Mantel-Cox) test at the *p* < 0.05 level. Open circles, fish injected with frgC_91–220_ spinycterins. Closed triangles, fish injected with Nmistic + frgC_91–220_ spinycterins. Closed circles, fish injected with YBEL + frgC_91–220_ spinycterins.

To preliminary experiment with some drugs to inactivate *E. coli* BL21(DE3) some quinolones (Ciprofloxacin, Levofloxacin, Oxolinic acid) and rifampicin were tested for colony formation of IPTG-induced YBEL + frgC_91–220_ spinycterins on 1.5% agar plates in TB medium containing ampicillin (A) and for preservation of the level of recombinant protein by polyacrylamide gel electrophoresis stained with Coomassie blue (B). The recombinant bacteria (3 × 10^10^ cfu/mL) were exposed during 2 h to 20 or 200 µg/mL of the selected quinolones and 10-fold lower concentrations of rifampicin. The plates were divided in two halves, plated with 5 (left) or 50 (right) µL of antibiotic-treated bacterial suspensions, incubated overnight at 37 °C and photographed. Red left arrow, YBEL + frgC_91–220_ recombinant protein.

Suspensions of 0.5 × 10^9^ cfu/mL of BLR(DE3) spinycterins in phosphate buffered saline (PBS) were agitated with a final concentration of 4% of paraformaldehyde for 20 h at 4 °C. Parallel suspensions of spinycterins were treated with PBS. The spinycterins suspensions were then quenched for 2 h with saturated glycine in PBS. Several concentrations of the resulting spinycterins were used to coat polyLys(D) 96-well plate solid-phases. (A), photomicrography of solid-phase bound spinycterins at 100 × 10^6^ cfu/mL (each bacteria length corresponded to ~2 µm). (B) The amount of exposed polyH tails was estimated by binding to peroxidase-conjugated anti-polyH monoclonal antibodies. The absorbance at 492 nM reflects the colour conversion of the OPD substrate. The absorbance at 620 nm was used to correct for individual well differences. Representative results of one of two experiments have been represented. Solid circles, YBEL + frgC_91–220_ spinycterins. Open circles, YBEL + frgC_91–220_ spinycterins treated with paraformaldehyde. Solid triangles, Nmistic + frgC_91–220_. Open triangles, Nmistic+frgC_91–220_ spinycterins treated with paraformaldehyde. Anti-polyH binding of spinycterins transformed with the empty pRSET plasmid showed Absorbances of ~0.5.

## Figures and Tables

**Table 1:** Fused to the *N*-terminus of frgC_91–220_ and resulting molecular weights.

Name	AccNum	KDa	References
frgC_91–220_	AY284969	16.2	[55]
Mistic + frgC_91–220_	AY874162	28.9	[35,56]
Nmistic + frgC_91–220_	AY874162	20.0	[35,56]
NTD + frgC_91–220_	AJ516945	18.4	[38]
P9 + frgC_91–220_	M12921	25.6	[24]
YAIN + frgC_91–220_	NP_414891	26.3	[39]
YBEL + frgC_91–220_	NP_415176	34.8	[39]

**Table 2:** Comparison of *E. coli* culture media.

Component	Concentration, %	TB	SB
Yeast extract	2.4	X	X
Glycerol	0.8	X	X
KHPO4	0.9	X	X
KH2PO4	0.2	X	X
Tryptone	1.2	X	--
Soybean hydrolysate	4.8	--	X
Glucose	0.3	--	X

Products were from Sigma Che. Co. (St.Louis, MS, USA).

## Supplementary Materials

The following are available online at https://www.mdpi.com/2076-393X/7/3/94/s1, Figure S1. Inhibition of colony formation of YBEL + frgC_91–220_ spinycterins by quinolones and rifampicin for *E. coli* BL21(DE3) inactivation (A) and preservation of recombinant protein levels (B). Figure S2. Spinycterins attachment to the solid-phase (A) and anti-polyH binding of Nmistic + frgC_91–220_ and YBEL + frgC_91–220_ spinycterins without or with paraformaldehyde (PFA) treatment (B).

## References

[1] Patel S., Korsnes K., Bergh O., Vik-Mo F., Pedersen J., Nerland A.H. (2007). Nodavirus in farmed Atlantic cod Gadus morhua in Norway. Dis. Aquat. Org..

[2] Gagne N., Johnson S.C., Cook-Versloot M., MacKinnon A.M., Olivier G. (2004). Molecular detection and characterization of nodavirus in several marine fish species from the northeastern Atlantic. Dis. Aquat. Org..

[3] ICTV International Committee on Taxonomy of Viruses (ICTV index of viruses). http://www.ncbi.nlm.nih.

[4] Munday B.L., Kwang J., Moody N. (2002). Betanodavirus infections of teleost fish: A review. J. Fish Dis..

[5] Skliris G.P., Krondiris J.V., Sideris D.C., Shinn A.P., Starkey W.G., Richards R.H. (2001). Phylogenetic and antigenic characterization of new fish nodavirus isolates from Europe and Asia. Virus Res..

[6] Nishizawa T., Furuhashi M., Nagai T., Nakai T., Muroga K. (1997). Genomic classification of fish nodaviruses by molecular phylogenetic analysis of the coat protein gene. Appl. Environ. Microbiol..

[7] Yong C.Y., Yeap S.K., Omar A.R., Tan W.S. (2017). Advances in the study of nodavirus. Peer J..

[8] Yamashita H., Fujita Y., Kawakami H., Nakai T. (2005). The efficacy of inactivated virus vaccine against viral nervous necrosis (NNV). Fish Patholol..

[9] Kai Y.H., Chi S.C. (2008). Efficacies of inactivated vaccines against betanodavirus in grouper larvae (Epinephelus coioides) by bath immunization. Vaccine.

[10] Lin C.S., Lu M.W., Tang L., Liu W.T., Chao C.B., Lin C.J., Krishna N.K., Johnson J.E., Schneemann A. (2001). Characterization of virus-like particles assembled in a recombinant baculovirus system expressing the capsid protein of a fish nodavirus. Virology.

[11] Thiery R., Cozien J., Cabon J., Lamour F., Baud M., Schneemann A. (2006). Induction of a protective immune response against viral nervous necrosis in the European sea bass Dicentrarchus labrax by using betanodavirus virus-like particles. J. Virol..

[12] Liu W., Hsu C.H., Chang C.Y., Chen H.H., Lin C.S. (2006). Immune response against grouper nervous necrosis virus by vaccination of virus-like particles. Vaccine.

[13] Husgard S., Grotmol S., Hjeltnes B.K., Rodseth O.M., Biering E. (2001). Irnmune response to a recombinant capsid protein of striped jack nervous necrosis virus (SJNNV) in turbot Scophthalmus maximus and Atlantic halibut Hippoglossus hippoglossus, and evaluation of a vaccine against SJNNY. Dis. Aquat. Org..

[14] Yuasa K., Koesharyani, Roza D., Mori K., Katata M., Nakai T. (2002). Immune response of humpback grouper, Cromileptes altivelis (Valenciennes) injected with the recombinant coat protein of betanodavirus. J. Fish Dis..

[15] Coeurdacier J.L., Laporte F., Pepin J.F. (2003). Preliminary approach to find synthetic peptides from nodavirus capsid potentially protective against sea bass viral encephalopathy and retinopathy. Fish Shellfish Immunol..

[16] Yamashita H., Mori K., Kuroda A., Nakai T. (2009). Neutralizing antibody levels for protection against betanodavirus infection in sevenband grouper, Epinephelus septemfasciatus (Thunberg), immunized with an inactivated virus vaccine. J. Fish Dis..

[17] Galeotti M., Romano N., Volpatti D., Bulfon C., Brunetti A., Tiscar P.G., Mosca F., Bertoni F., Marchetti M.G., Abelli L. (2013). Innovative vaccination protocol against vibriosis in Dicentrarchus labrax (L.) juveniles: Improvement of immune parameters and protection to challenge. Vaccine.

[18] Huang S.M., Cheng J.H., Tu C., Chen T.I., Lin C.T., Chang S.K. (2017). A bivalent inactivated vaccine of viral nervous necrosis virus and grouper iridovirus applied to grouper broodfish (Epinephelus coioides) reduces the risk of vertical transmission. Taiwan Vet. J..

[19] Buonocore F., Nunez-Ortiz N., Picchietti S., Randelli E., Stocchi V., Guerra L., Toffan A., Pascoli F., Fausto A.M., Mazzini M. (2019). Vaccination and immune responses of European sea bass (Dicentrarchus labrax L.) against betanodavirus. Fish Shellfish Immunol..

[20] Gonzalez-Silvera D., Guardiola F.A., Espinosa C., Chaves-Pozo E., Esteban M., Cuesta A. (2019). Recombinant nodavirus vaccine produced in bacteria and administered without purification elicits humoral immunity and protects European sea bass against infection. Fish Shellfish Immunol..

[21] Coll J.M. (2017). Fish mass immunization against virus with recombinant “spiny” bacterins. Fish Shellfish Immunol..

[22] Chen C.W., Wu M.S., Huang Y.J., Cheng C.A., Chang C.Y. (2015). Recognition of Linear B-Cell Epitope of Betanodavirus Coat Protein by RG-M18 Neutralizing mAB Inhibits Giant Grouper Nervous Necrosis Virus (GGNNV) Infection. PLoS ONE.

[23] Costa J.Z., Adams A., Bron J.E., Thompson K.D., Starkey W.G., Richards R.H. (2007). Identification of B-cell epitopes on the betanodavirus capsid protein. J. Fish Dis..

[24] Jung Y., Jung H., Lim D. (2015). Bacteriophage membrane protein P9 as a fusion partner for the efficient expression of membrane proteins in *Escherichia coli*. Protein Expr. Purif..

[25] Molloy M.P., Herbert B.R., Slade M.B., Rabilloud T., Nouwens A.S., Williams K.L., Gooley A.A. (2000). Proteomic analysis of the *Escherichia coli* outer membrane. Eur. J. Biochem..

[26] Jeong K.S., Xie Y., Hiasa H., Khodursky A.B. (2006). Analysis of pleiotropic transcriptional profiles: A case study of DNA gyrase inhibition. PLoS Genet..

[27] Schmidt M., Romer L., Strehle M., Scheibel T. (2007). Conquering isoleucine auxotrophy of *Escherichia coli* BLR(DE3) to recombinantly produce spider silk proteins in minimal media. Biotechnol. Lett..

[28] Goffin P., Dehottay P. (2017). Complete Genome Sequence of *Escherichia coli* BLR(DE3), a recA-Deficient Derivative of *Escherichia coli* BL21(DE3). Genome Announc..

[29] Bovo G., Nishizawa T., Maltese C., Borghesan F., Mutinelli F., Montesi F., De Mas S. (1999). Viral encephalopathy and retinopathy of farmed marine fish species in Italy. Virus Res..

[30] Baudin-Laurencin F., Richards R. (1999). Nodavirus Workshop. Bull. Eur. Assoc. Fish Pathol..

[31] Chen N.C., Yoshimura M., Guan H.H., Wang T.Y., Misumi Y., Lin C.C., Chuankhayan P., Nakagawa A., Chan S.I., Tsukihara T. (2015). Crystal Structures of a Piscine Betanodavirus: Mechanisms of Capsid Assembly and Viral Infection. PLoS Pathog..

[32] Petrovskaya L.E., Shulga A.A., Bocharova O.V., Ermolyuk Y.S., Kryukova E.A., Chupin V.V., Blommers M.J., Arseniev A.S., Kirpichnikov M.P. (2010). Expression of G-protein coupled receptors in *Escherichia coli* for structural studies. Biochemtry.

[33] Blain K.Y., Kwiatkowski W., Choe S. (2010). The functionally active Mistic-fused histidine kinase receptor, EnvZ. Biochemistry.

[34] Nekrasova O.V., Wulfson A.N., Tikhonov R.V., Yakimov S.A., Simonova T.N., Tagvey A.I., Dolgikh D.A., Ostrovsky M.A., Kirpichnikov M.P. (2010). A new hybrid protein for production of recombinant bacteriorhodopsin in *Escherichia coli*. J. Biotechnol..

[35] Roosild T.P., Greenwald J., Vega M., Castronovo S., Riek R., Choe S. (2005). NMR structure of Mistic, a membrane-integrating protein for membrane protein expression. Science.

[36] Dvir H., Lundberg M.E., Maji S.K., Riek R., Choe S. (2009). Mistic: Cellular localization, solution behavior, polymerization, and fibril formation. Protein Sci..

[37] Dvir H., Choe S. (2009). Bacterial expression of a eukaryotic membrane protein in fusion to various Mistic orthologs. Protein Expr. Purif..

[38] Park T.J., Heo N.S., Yim S.S., Park J.H., Jeong K.J., Lee S.Y. (2013). Surface display of recombinant proteins on *Escherichia coli* by BclA exosporium of *Bacillus anthracis*. Microb. Cell Fact..

[39] Leviatan S., Sawada K., Moriyama Y., Nelson N. (2010). Combinatorial method for overexpression of membrane proteins in *Escherichia coli*. J. Biol. Chem..

[40] Studier F.W. (2005). Protein production by auto-induction in high density shaking cultures. Protein Expr. Purif..

[41] Studier F.W. (2014). Stable expression clones and auto-induction for protein production in *Escherichia coli*. Methods Mol. Biol..

[42] Xu J., Banerjee A., Pan S.H., Li Z.J. (2012). Galactose can be an inducer for production of therapeutic proteins by auto-induction using *Escherichia coli* BL21 strains. Protein Expr. Purif..

[43] Somrit M., Watthammawut A., Chotwiwatthanakun C., Ounjai P., Suntimanawong W., Weerachatyanukul W. (2017). C-terminal domain on the outer surface of the Macrobrachium rosenbergii nodavirus capsid is required for Sf9 cell binding and internalization. Virus Res..

[44] Sivashanmugam A., Murray V., Cui C., Zhang Y., Wang J., Li Q. (2009). Practical protocols for production of very high yields of recombinant proteins using *Escherichia coli*. Protein Sci..

[45] Collin F., Karkare S., Maxwell A. (2011). Exploiting bacterial DNA gyrase as a drug target: Current state and perspectives. Appl. Microbiol. Biotechnol..

[46] Kohanski M.A., Dwyer D.J., Hayete B., Lawrence C.A., Collins J.J. (2007). A common mechanism of cellular death induced by bactericidal antibiotics. Cell.

[47] Silva F., Lourenco O., Queiroz J.A., Domingues F.C. (2011). Bacteriostatic versus bactericidal activity of ciprofloxacin in *Escherichia coli* assessed by flow cytometry using a novel far-red dye. J. Antibiot..

[48] Mustaev A., Malik M., Zhao X., Kurepina N., Luan G., Oppegard L.M., Hiasa H., Marks K.R., Kerns R.J., Berger J.M. (2014). Fluoroquinolone-gyrase-DNA complexes: Two modes of drug binding. J. Biol. Chem..

[49] Gillund F., Kjolberg K.A., von Krauss M.K., Myhr A.I. (2008). Do uncertainty analyses reveal uncertainties? Using the introduction of DNA vaccines to aquaculture as a case. Sci. Total Environ..

[50] Zhou Y.C., Wang J., Zhang B., Su Y.Q. (2002). Ultrasonic immunization of sea bream, Pagrus major (Temminck & Schlegel), with a mixed vaccine against Vibrio alginolyticus and V. anguillarum. J. Fish Dis..

[51] Navot N., Sinyakov M.S., Avtalion R.R. (2011). Application of ultrasound in vaccination against goldfish ulcer disease: A pilot study. Vaccine.

[52] Cobo C., Makosch K., Jung R., Kohlmann K., Knop K. (2013). Enhanced bacterin permeability and side effects using low frequency sonophoresis at 37 kHz in rainbow trout. Fish Shellfish Immunol..

[53] Labarca C.C., Makhutu M., Lumsdon A.E., Thompson K.D., Jung R., Kloas W., Knopf K. (2015). The adjuvant effect of low frequency ultrasound when applied with an inactivated Aeromonas salmonicida vaccine to rainbow trout (Oncorhynchus mykiss). Vaccine.

[54] Torrealba D., Parra D., Seras-Franzoso J., Vallejos-Vidal E., Yero D., Gibert I., Villaverde A., Garcia-Fruitos E., Roher N. (2016). Nanostructured recombinant cytokines: A highly stable alternative to short-lived prophylactics. Biomaterials.

[55] Ucko M., Colorni A., Diamant A. (2004). Nodavirus infections in Israeli mariculture. J. Fish Dis..

[56] Alves N.S., Astrinidis S.A., Eisenhardt N., Sieverding C., Redolfi J., Lorenz M., Weberruss M., Moreno-Andres D., Antonin W. (2017). MISTIC-fusion proteins as antigens for high quality membrane protein antibodies. Sci. Rep..

