# Supplementary material for: Sea Bass Immunization to Downsize the Betanodavirus Protein Displayed in the Surface of Inactivated Repair-Less Bacteria
Source: Vaccines (Basel). 2019 Aug 20;7(3):94. doi: 10.3390/vaccines7030094 (PMC6789578; doi:10.3390/vaccines7030094)
Supplement: Supplementary file 1 [file vaccines-07-00094-s001.pdf]

Supplementary

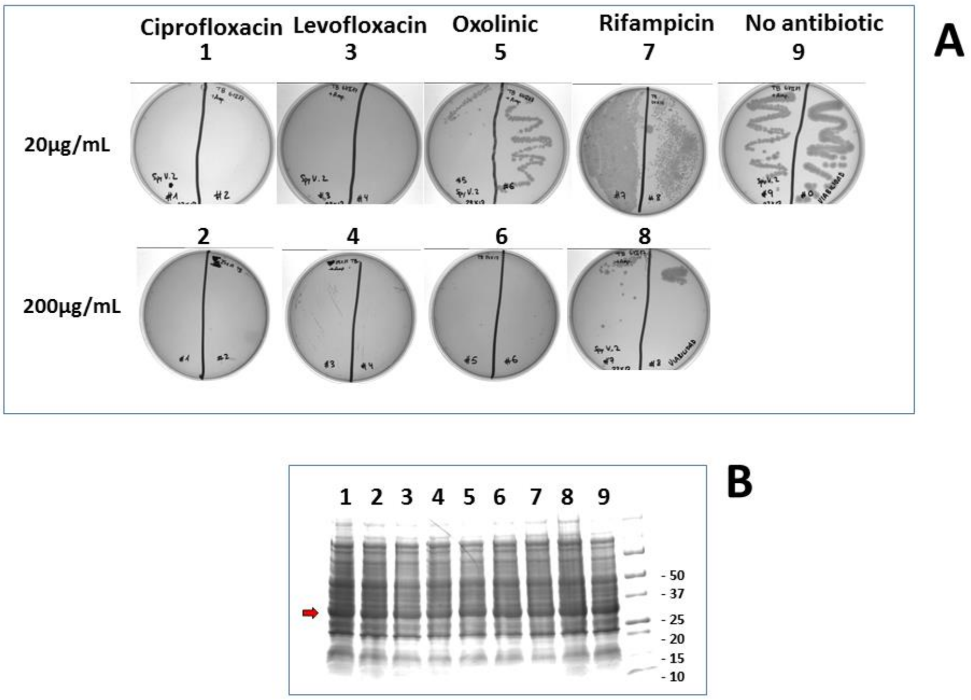

Figure S1. Inhibition of colony formation of YBEL+frg$C_{91–220}$ spinycterins by quinolones and rifampicin for *E. coli* BL21(DE3) inactivation (**A**) and preservation of recombinant protein levels (**B**).

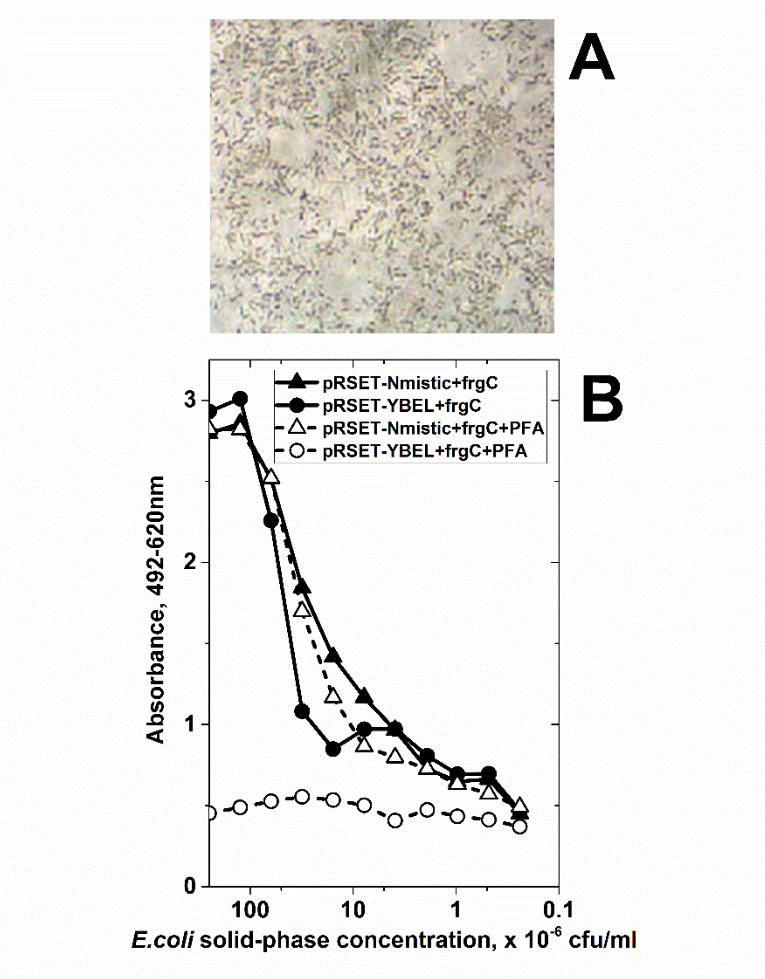

Figure S2. Anti-polyH binding of Nmistic+frgC$_{91–220}$ and YBEL+frgC$_{91–220}$ spinycterins without or with paraformaldehyde (PFA) treatment.
